# Screen use in children – two sides of the coin: a critical narrative review

**DOI:** 10.1590/1980-5764-DN-2024-0173

**Published:** 2025-03-17

**Authors:** Everton Ferreira de Souza, Rafael Antônio Vicente Lacerda, Janaína Aparecida Favero Desio, Camila Marciele Kammers, Silvana Henkes, Natasha Ferraz da Paixão Ribeiro, Monique Freitas de Sá, Driele Martins da Silva, Anna Carolina de Oliveira Resende Teixeira, Júlio César Claudino dos Santos

**Affiliations:** 1 Universidade Federal de Juiz de Fora, Faculdade de Medicina, Juiz de Fora MG, Brazil.; 2 Faculdade de Medicina do Juazeiro de Norte, Departamento de Medicina Interna, Juazeiro do Norte CE, Brazil.; 3 Centro Universitário Nossa Senhora do Patrocínio, Programa de Pós-Graduação em Psicologia, Itu SP, Brazil.; 4 Universidade Estadual do Norte do Paraná, Jacarezinho PR, Brazil.; 5 Universidade Luterana do Brasil, Carazinho RS, Brazil.; 6 Universidade Estácio de Sá, Programa de Pós-Graduação em Psicologia, Rio de Janeiro RJ, Brazil.; 7 Universidade Potiguar, Natal RN, Brazil.; 8 Universidade Federal Rural do Rio de Janeiro, Rio de Janeiro RJ, Brazil.; 9 Istituto de Pós-Graduação e Graduação, Goiânia GO, Brazil.; 10 Centro Universitário Christus, Faculdade de Medicina, Fortaleza CE, Brazil.; 11 Universidade Federal do Ceará, Departamento de Morfologia, Programa de Pós-Graduação em Ciências Morfofuncionais, Fortaleza CE, Brazil.; 12 Centro Universitário Facvest, Lages SC, Brazil.

**Keywords:** Screen Time, Environment, Child Guidance, Child Development, Tempo de Tela, Meio Ambiente, Orientação Infantil, Desenvolvimento Infantil

## Abstract

Like a coin, the impacts of screens on children’s development have two sides, as reflected in current scientific knowledge. This narrative review aimed to explore the dual-faceted state of the art regarding screen use, highlighting both positive and negative aspects on neurodevelopment, intervention proposals, and future perspectives for appropriate screen use. Recent scientific findings emphasize two central points: on the one hand, the benefits of appropriate use, such as co-viewing and the use of educational content; on the other hand, the negative impacts of excessive screen use, passive, and non-educational use on the development of neural networks. In this sense, with the advent of the digital age and in light of current scientific results, it is clear that eliminating screens from daily life is unrealistic. Therefore, implementing strategies to ensure a healthy balance between screen time and other activities important for child development is essential.

## RESUMO

Como uma moeda, à luz do conhecimento científico atual, pode-se dizer que os impactos das telas no desenvolvimento das crianças possuem dois lados. Esta revisão narrativa teve como objetivo explorar o bifacetado estado da arte acerca do uso de telas, evidenciando pontos positivos e negativos no neurodesenvolvimento, propostas de intervenções e perspectivas futuras para adequação no uso das telas. Achados científicos recentes evidenciam dois pontos centrais: por um lado, os benefícios do uso adequado, como a covisualização e o uso de conteúdos educacionais. Por outro lado, os impactos negativos do uso excessivo de telas, de maneira passiva e não educativa, no desenvolvimento das redes neurais. Nesse sentido, com o advento da era digital e de posse dos resultados científicos atuais, é evidente que eliminar as telas da vida cotidiana é irrealista. Portanto, é essencial implementar estratégias que garantam um equilíbrio saudável entre o tempo de tela e outras atividades importantes para o desenvolvimento infantil.

## INTRODUCTION

From gestation to the first five years of life, the human brain undergoes rapid transformations, modulated by genetic activation and adaptation to environmental stimuli^1,2,3,4^. These early years are crucial for neuropsychomotor development (NPMD) in an organized manner and the replacement of reflexes with more complex movements, as well as presenting significant individual variability in the speed of skill acquisition^5,6,7,8^.

Given this window of accelerated nervous system development, exposure to screens can have notable impacts — both positive and negative^9,10,11,12^. Studies suggest that excessive screen time may negatively affect areas such as language development, attention, and social skills, as well as poor sleep and behavioral disturbance, which are intensely developed during early childhood^9,10,11,12,13,14,15,16^. On the other hand, while background television has been shown to have negative effects, educational programs and co-viewing can yield positive outcomes, such as improved language skills^9^. In this sense, scientific evidence supports pediatric recommendations to limit the duration of children’s screen exposure, select high-quality programming, and engage in co-viewing whenever possible^17,18,19^.

In 2016, the American Academy of Pediatrics (AAP) recommended avoiding screen time for children under two years of age (except for video chatting) and limiting screen use to one hour per day for children aged two to five^20^. Similar guidelines have been adopted by the World Health Organization^21^, as well as pediatric societies around the world (e.g., Canadian Guidelines and Australian Guidelines). In this sense, excessive screen use is generally defined as prolonged and frequent use of electronic devices, such as smartphones, computers, and tablets, exceeding daily exposure time recommendations^12,22^. Children and adolescents now experience increasing exposure and access to digital media, reaching seven hours of usage a day^13^. This trend has proved even more relevant during the COVID-19 pandemic, where there has been an increase in overall screen exposure time, leading to greater disruption in the mental well-being of children and adolescents, as assessed by various studies^12,23,24,25,26^.

Children under five are the fastest-growing group of digital media users, yet few adhere to recommended screen time guidelines, with an average daily screen time of approximately seven hours^9,13,27^. With this in mind, since the use of digital devices is unanimous and growing in the population, interventions aimed at reducing screen exposure, such as promoting outdoor play^28^, and mediations that promote co-assistance with family members and educational programs^29,30^, should be encouraged in order to reduce the negative impact on children’s brain development, taking into account that total restriction is unrealistic, especially due to socioeconomic factors^31,32,33^.

Given the global increase in excessive screen use by children, especially in early childhood, this critical narrative review aimed to investigate the potential harm and benefits of screen use on neurodevelopment and to explore interventions and measures aimed at reducing screen time.

## NEUROBIOLOGICAL AND FUNCTIONAL ASPECTS ASSOCIATED WITH EXPOSURE TO SCREENS

The idea of an immutable brain has long been deconstructed^34^. Neuroplasticity is based on the changes in the dynamics of neuronal groups due to the influence of various environmental factors, allowing a better understanding of adaptive complexity, vulnerability, and brain disruption^35,36^. In this context, much has been discussed about the relationship between screen time in childhood and the increased risk of neurodevelopmental disorders^37,38^.

A study correlating neurobiological structure to screen exposure found that prolonged screen time was associated with reduced anisotropy and increased radial diffusivity in the arcuate, uncinate, and inferior longitudinal fasciculi, with a predominance of left lateralization. These fiber bundles are known to be associated with language and literacy, in terms of orthographic-phonological translation, semantic processing, emotional regulation, and visual representation formation^39^.

Recent studies using resting-state functional magnetic resonance imaging (fMRI) have demonstrated that prolonged screen exposure reduced frontostriatal activity, predicting a delay in the development of inhibitory control. Additionally, there was also a negative modulation of frontostriatal connectivity, amplifying reward sensitivity^40^. Similarly, research involving cannabis-dependent individuals revealed lower connectivity between the striatum and frontal regions compared to non-addicted participants^41^. Thus, it is possible that prolonged screen exposure could result in changes in brain connectivity resembling those observed in compulsive chemical-seeking behaviors^42^.

When comparing executive functions according to screen exposure, on one hand, it was not evidenced that executive function worsened over time in the high-exposure group compared to the control group^43^. On the other hand, an electroencephalogram (EEG) study involving 437 children at nine years of age, who had engaged in excessive screen use at 12 months of age, showed that using screens for more than four hours per day increased theta wave activity and the theta/beta ratio in the frontoparietal regions. This suggests that the mechanism mediated by the frontoparietal pathway in excessive screen use is responsible for the worsening of attention and working memory^44^.

However, scientific outcomes remain conflicting, especially when correlations are individualized with the nature of digital content and the way it is accessed, for example, touch screen *versus* passive viewing, influencing different aspects of brain function. Research using functional spectroscopy observed that the addition of tactile feedback during hyper-stimulating events reduced hyperactivation of the dorsolateral prefrontal cortex, mitigating the increased cognitive demand and impaired inhibitory control observed in passive viewing^45^. Furthermore, studies employing electroencephalography through event-related potentials (EEG/ERP) during video game interaction demonstrated increased beta and theta waves activity in the frontal lobe; the former correlating with memory acquisition processes, while the latter with learning^46^. These findings reflect improved cognitive performance, working memory, and information processing, in addition to differences in key regions of the cortex responsible for vision and attention^47^.

Additionally, parent-child reading serves as a protective factor, potentially mitigating the adverse effects of screen time on brain network integration and socioemotional development^29^. In this regard, a cohort study with nearly 12,000 adolescents (aged 9 to 12 years old) from the Adolescent Brain Cognitive Development (ABCD) study evaluated screen use and its association with patterns of functional connectivity and neural development. Despite the high screen time of 26.50 hours per week, there was no association with changes in connectomes and patterns of functional brain organization during the study period. Even with subsequent analyses of the trajectories and maturation of neural networks over two years, no association was observed^48^.

It is thus essential to recognize the importance of continuing studies in this scope, given the complexity of interactions between screen use and children’s brain development. A deeper understanding of the effects of early exposure to digital devices is crucial for identifying clear patterns and potential long-term consequences. Furthermore, developing effective strategies to track and mitigate early neurodevelopmental changes is crucial to ensuring that children can benefit from digital technologies in a safe and healthy manner. This involves not only understanding the direct impacts of screen exposure on the brain but also considering contextual factors, such as the nature of digital content and how it is consumed. By promoting more conscious and balanced use of technology from childhood, we can contribute to healthy and promising brain development for future generations.

## BENEFITS OF PROPER SCREEN USE FOR CHILDREN: ONE SIDE OF THE COIN

In addition to age range and usage time, other factors are associated with the benefits of screens in children’s development. For example, the type of media (TV, DVD, smartphone, PC, or video games) plays a significant role^49^. Certain types of games promote physical well-being by encouraging children to run, jump, and engage in other physical activities. Additionally, a recent study by Yang et al.^50^ involving 120 children at risk of dyslexia who trained with action video games demonstrated specific, yet distant, phonological transfer benefits compared to conventional treatments.

A French national cohort study^51^, which followed 13,763 infants from 2 months to 5.5 years old, found that the association between screen use and cognitive development at 2 years of age follows an inverted U-curve but is linear at 3.5 and 5.5 years old. Children with no screen exposure had lower cognition scores compared to those exposed in a controlled manner (<1 hour/day) and interactively with their parents. At 3.5 years, intermediate screen use at 2 years was associated with a positive effect on non-verbal reasoning, leading to improved cognitive performance^49,51^.

Supporting the importance of context in screen use, data from the Language Environment Analysis (LENA) device evaluated 220 Australian families. At 12 months old, babies were exposed to an average of 87.8 minutes of screens per day, heard 14,997 words from adults, produced 1,394 vocalizations, and engaged in 369.4 conversational turn-taking exchanges. Conversely, even on families following WHO recommendations of 1 hour of screen use per day at 36 months, children experienced a reduction of 397 words spoken by adults, 294 vocalizations, and 68 turn-taking exchanges^21,52^. Parent interaction during screen use provides high-quality content (educational programs or interactive games), enhancing children’s language and cognition^53^. Interactive screen use with parents can increase a child’s vocabulary and literacy skills^54^.

Regarding screen content, another crucial factor is the cultural context of what is watched. Passive learning is less effective, but parental mediation and explanation can foster better development^49,55^. High-quality educational TV programs can serve as an additional means for early language and literacy development, cognitive development, promoting positive racial attitudes, and encouraging imaginative play^56^. For children with neurodevelopmental disorders (NDDs) such as attention deficit hyperactivity disorder (ADHD), dyslexia, and dyscalculia, digital game-based training showed lasting effects (3 to 9 weeks post-intervention) and effectively trained cognitive skills. This training can be replicated at home^57^.

A meta-analysis of 18,905 children up to 12 years old found small to moderate effects on language skills when educational content was viewed, parental interaction was present, and screen introduction occurred at older ages^9^. Screen use can have both positive and negative outcomes in NDDs. Social media use may benefit children with autism spectrum disorder (ASD) symptoms by engaging in interpersonal associations. Certain video games are positively associated with intellectual functioning and academic performance and have been developed to provide emotional support and joyful experiences for children with ASD^58,59^.

To better understand the age factor in social skills, a US national cohort evaluated 2,152 babies at 12 and 18 months old, using the M-CHAT and M-CHAT-R scales at 2 years old to investigate autism risk and autism-like manifestations, respectively. Passive TV/video use at 12 months (but not at 18 months) was associated with autism-like manifestations at 2 years old by M-CHAT-R (change, 4.2%), but not autism risk by M-CHAT (risk prevalence rates, 8.3 *vs*. 4.4%)^60^. It is important to note that screen use does not cause autism.

Regarding executive functions, a Canadian cohort evaluated 2,983 children, finding that 39.7% spent more than one hour per day on screens. Preschoolers who followed the recommendation of up to 1 hour/day had better working memory performance^61^, and educational programs were associated with improved inhibitory control^62^. In contrast, a cross-sectional study in Ceará, Brazil, with 3,155 children aged 0 to 60 months, found that each additional hour beyond the recommended screen time was associated with poorer child communication skills^63^. Controlled exposure to interactive media can enhance cognitive development, facilitate learning, develop visual and motor skills, and promote creativity, problem-solving, and social interaction^29,40,64^.

Finally, in addition to the benefits of certain types of media, interactive use with parents, and educational programs considering cultural context, age limits, and screen time, it appears that the female gender may protect against the negative impacts of improper screen use. In a US cohort of 101,350 children up to 17 years old, no significant negative effects were observed in girls across any age group^65^.

## THE DRAWBACKS OF SCREEN USE: THE OTHER SIDE OF THE COIN

The increasing prevalence of electronic devices and their intensified use by children raise significant concerns about their adverse impacts on development. Excessive screen time is known to negatively contribute to physical development and contribute to sleep problems and obesity, especially among school-aged children and adolescents^66,67,68,69^. A national cohort study in the United States, conducted between 2018 and 2020, evaluated nearly 103,000 children and young people up to 17 years of age from the National Survey of Children’s Health (NSCH) regarding developmental delays associated with excessive screen use. The delays were more pronounced in preschool boys who used screens for more than 4 hours a day (OR 2.12). Specifically, the risk of communication delays was more than doubled (OR 2.38)^65^. Conversely, a Japanese cohort study involving 7,097 children up to 4 years old found that screen use of more than 4 hours per day before the first year of life increased the risk of communication delays by 4.7 times (OR 4.78) and more than doubled the risk in the second year of life for problem-solving (OR 2.67) and social skills delays (OR 2.10). Communication delays persisted until the age of 4 (OR 2.68)^70^.

Regarding the impacts of excessive screen use on language, a recent systematic review found that excessive screen time before the age of 2 can negatively impact language development and communication skills^49^. Also, children who watched TV without parental interaction were found to have an 8.47-fold increase in the risk of language acquisition delays. TV use during family meals was shown to hinder expressive language development in children aged 2 to 6 years, leading to reduced vocabulary, comprehension, and gestural production with communicative intent^49,51^. Using the LENA device, there was a reduction of more than 500 to 1,000 words per day spoken by parents to their children and 60 to 200 conversational turn-taking exchanges, even when following WHO recommendations for screen use^49,52^.

The practicality and widespread availability of mobile media devices appear to contribute to a growing emotional dysregulation among children during a critical phase of neurodevelopment. A prospective study involving 315 preschool-aged children (3.5 to 5.5 years old) observed a negative bidirectional relationship in the screens-executive functions dynamic. At age 3.5, an increase in tablet use by 1.22 hours per day was associated with a 22% increase in the standard deviation (SD) of expressions of anger and frustration at age 4.5 (standardized coefficient=0.22; 95%CI 0.01–0.44). Conversely, worsening expressions of anger at age 4.5 led to an increase of 0.28 hours of additional tablet use at age 5.5 for each 1 SD increase in the Children’s Behavior Questionnaire (standardized coefficient=0.22; 95%CI 0.01–0.43), thus highlighting a vicious and detrimental cycle during a critical period for the development of executive functions and emotional regulation^71^. Similarly, corroborating previous findings, another recent study involving preschoolers with a mean age of 3.8 years revealed that, after three and six months of assessment, boys whose parents used mobile devices to calm them exhibited poorer emotional reactivity (r=0.20). Furthermore, as a feedback loop, this poorer emotional reactivity resulted in an increased use of devices for calming (r=0.13)^72^. Therefore, there is growing evidence that parental practices aimed at emotional suppression of children through media use may not only reinforce inappropriate use of mobile devices by children but also establish a paradoxical regulatory effect on executive functions in the medium and long term^71,72^.

A study involving approximately 11,000 participants in the United States found that excessive TV, video, and video game use was associated with decreased sleep duration, increased time to fall asleep, and intensified symptoms of sleep-wake disorders, such as insomnia and excessive sleepiness^73^. Other research supports these findings, showing that high levels of screen exposure result in lower sleep efficiency, delayed sleep onset, increased frequency of night awakenings, and greater daytime sleepiness compared to lower exposures^67,68,74^. The underlying mechanisms of screen impact on sleep include exposure to blue light, which increases alertness and suppresses melatonin production, delaying sleep onset^66,67,73,75,76^.

Additionally, excessive screen use promotes a more sedentary lifestyle among children and adolescents, leading to reduced physical activity and lower energy expenditure, which can negatively affect sleep quality and weight gain^67,74^. According to the World Health Organization, it is recommended that children and adolescents aged 5 to 17 engage in at least 60 minutes of moderate to vigorous physical activity daily. This should include intense aerobic exercises and activities that strengthen muscles and bones at least three times a week^21,77,78^. This recommendation is crucial since children under 5 years old spend 50% of their time engaged in sedentary behaviors^54,79^.

In addition to the negative effects of sedentary behavior, excessive screen use is linked to increased body mass index (BMI) in children, which raises the risk of developing conditions such as high blood pressure, elevated insulin levels, and high cholesterol during adolescence^69^. A longitudinal study conducted in Sweden with children and adolescents aged 4 to 17 years, between 2018 and 2021, identified interesting trends in behavior related to physical activity and screen use^80^. It was observed that as children grow, physical activity generally decreases, with girls consistently showing lower activity levels compared to boys. Simultaneously, there was a significant increase in screen time, which peaked in 2020, coinciding with the COVID-19 pandemic. During the pandemic, not only did screen use increase, but physical activity among this age group decreased^80,81,82^.

An increased risk of obesity has been observed in children who spend excessive time in front of screens. This phenomenon can be explained by the replacement of physical activity with more sedentary behaviors associated with digital device use. Additionally, inadequate eating patterns, including increased calorie intake and higher frequency of fast food and sweets consumption, as well as decreased intake of fiber, vegetables, fruits, and fish, are often found among young people who spend excessive time on screens. Studies suggest that prolonged screen use can contribute to higher calorie consumption, as it distracts attention during meals, leading to less control over what and how much is eaten^81,82,83^.

It is essential to raise awareness about the dangers of excessive screen use for children’s health, emphasizing the importance of adequate sleep for their development, physical activity practices, and a healthy and balanced diet. This requires a joint effort from health professionals, educators, and public policies, along with guidance and support for parents in promoting conscious screen use at home.

## EXCESSIVE SCREEN USE AND SOCIOECONOMIC STATUS

In a society where race and socioeconomic status determine not only opportunities and treatment but also health outcomes^84,85,86,87,88,89^, it is vital that investigations are conducted to evaluate the association between socioeconomic status, race, and excessive screen time among children. According to Nagata et al.^14^, in a study analyzing the sociodemographic data and screen use of 10,775 children aged 9 and 10 years, the average screen time was 3.99 hours per day. However, black children reported 1.58 more hours of screen time, while Asian children reported 0.35 fewer hours of screen time, both compared to white children who had an average of 3.46 hours of screen time per day. Similar results were found in other studies^12,85,89,90,91^.

For instance, another cross-sectional study with a sample of 48,775 children aged 6 months to 5 years found an increase in screen use during the first year of the COVID-19 pandemic, which returned to pre-pandemic levels in 2021. However, the increase in screen time remained elevated among children living in poverty, with nearly 3 out of 4 black children still experiencing high screen time in 2021^12^. Moreover, there is an inverse relationship between family income and children’s screen time^14,89^. In the study by Assari^89^, with 15,022 children aged 9 to 11 years to evaluate the association between screen time, family income, and race, it was concluded that black, mixed-race, or children of other races had the lowest levels of education and family income, placing them at a higher risk for excessive screen use and its associated harms. Lower parental education was also associated with increased total screen time, video watching, and texting^14^.

However, another important point to consider in the discussion is that this inverse association between family income and screen time was more relevant among white children. Due to limited access to opportunities for physical activity and sports, inadequate access to safe recreational areas, and the impact of structural and systematic discrimination, black families live under intense stress regardless of their socioeconomic status, reducing the impact that family income has on decreasing screen time, a factor not observed in white families^86,87,88^. Given the significant sociodemographic differences in children’s screen use, it is necessary to develop specific strategies and guidelines for healthy screen use for these vulnerable groups. This approach will help reduce the gap in opportunities and unsatisfactory health outcomes between different ethnic-racial groups and those with varying socioeconomic statuses.

## INTERVENTION PROPOSALS AND FUTURE PERSPECTIVES

In this scope, as part of a health policy, AAP recommends the development of a *Family Media Plan* to establish rules for screen use at home based on a series of topics aligned with the entity’s recommendations. Among these is the creation of a “screen-free zone,” referring to areas where screen use should be avoided, such as the bedroom and during meals, to allow for parent-child interaction during media exposure^20,92^. Additionally, another recommendation aims to mitigate the negative effects of screens on sleep, such as restricting screen use at least one hour before bedtime and avoiding associated sedentary behavior by encouraging activities outside of screen time^93^. Moreover, it is important to reduce unintentional screen use. There are two avoidable pitfalls in this regard. First, using screens as a digital babysitter limits the physical presence of parents during exposure^49^. Second, screens should not be used as a means to suppress a child’s tantrums or crying, as this can harm the development of executive functions, self-control, and emotional regulation^72^.

Given the issues to screen use, many caregiver practices are directly associated with children’s screen use. Habit formation during childhood is influenced by caregivers’ experiences with media^52,69,72,79,94^. Beyond advising on current screen time guidelines, a “harm reduction” approach with intervention proposals would be more effective, especially for children under 5 years old^79,95^. Strategies focused on children over 6 years old aim to recognize the early signs of harmful screen use^96^. To capture the spectrum of possible problems in this scenario, the acronym PIMU, for problematic interactive media use, was developed in 2019^16^. In this context, the importance of behavioral therapies, such as cognitive and dialectical, as well as motivational enhancement therapy, focused on the triad of “Goals, Feedback, and Planning,” with an average duration of 4 to 12 months, is established^97,98,99,100^.

Today, there is still a need for future research with better capabilities to measure screen time using sensitive devices such as the LENA device^52,100,101,102,103^. Furthermore, it is essential to consider variables such as the mental health status of caregivers and to include sociodemographic factors in analyses, given the increase in screen use, especially among children from low socioeconomic status families^70,104,105,106^. To this end, an integrated socioecological model has recently been proposed, encompassing environmental variables, caregivers’ health, stress, self-regulation, and socioeconomic status^79^.

Digital training intervention therapies using games for children with NDDs, whether ADHD, dyslexia, or dyscalculia, appear to be a promising area for further exploration^11^. Additionally, the benefits of using Artificial Intelligence (AI) in children’s development and learning should be explored. Proposals have been made to reconstruct early childhood education with a curriculum based on three central questions: *Why use AI? What type of AI? How to engage in learning AI concepts?* ^107,108,109^. This model proposes AI literacy as an organic part of digital literacy for all citizens in a vibrant and developed society^110,111,112^.

**Figure 1:**
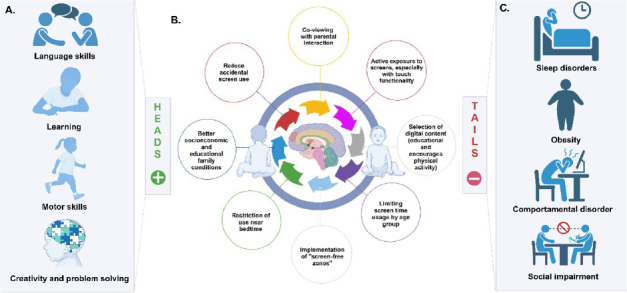
Graphical abstract. “Flip a coin”: the two sides of the coin of controlled exposure and excessive exposure to screens in children’s neurodevelopment. A. Heads: Benefits of proper screen use for children. B. Protective interventions in screen use for the neurobiology of child development. C. Tails: The drawbacks of excessive screen use for children.

In summary, this narrative review highlights the dual-faceted impact of screen use on children’s neurodevelopment (Figure 1). On one side of the coin, controlled and educational screen use, utilizing protective factors, can offer cognitive benefits and enhance creativity, improve language and motor skills, as well as assist in learning. Conversely, excessive and unregulated screen use is associated with detrimental effects, including the development of behavioral disorders, particularly dysregulation of hot executive functions related to anger and frustration, sleep disturbances, increased risk of obesity, and impairment of socialization. For a quick analysis of the main evidence clarifying this dual impact, refer to Supplementary Material Table S1 (available at https://www.demneuropsy.com.br/wp-content/uploads/2024/12/DN-2024.0173-Supplementary-Material.docx).

The findings emphasize the importance of implementing balanced and mindful screen use strategies. Given the ubiquity of digital media in contemporary life, eliminating screen exposure is impractical. Instead, targeted interventions, such as implementing “screen-free” zones, encouraging outdoor activities, and promoting high-quality educational content, are essential to mitigate the adverse effects while harnessing the potential benefits of screens.

Future research should continue to explore the nuanced interactions between screen time and neurodevelopment, considering variables like content type, context of use, and sociodemographic factors. By fostering a comprehensive understanding and promoting informed screen use practices, we can support healthy brain development and well-being in children amidst the digital age.
